# Variability of surface and underwater nocturnal spectral irradiance with the presence of clouds in urban and peri-urban wetlands

**DOI:** 10.1371/journal.pone.0186808

**Published:** 2017-11-08

**Authors:** Jean Secondi, Valentin Dupont, Aurélie Davranche, Nathalie Mondy, Thierry Lengagne, Marc Théry

**Affiliations:** 1 UMR 5023 Université de Lyon, Écologie des Hydrosystèmes Naturels et Anthropisés, Université Lyon 1, ENTPE, CNRS, Villeurbanne, France; 2 GECCO, Université Angers, France; 3 UMR CNRS 6554 LETG-LEESA, Université d’Angers, France; 4 UMR 7179 CNRS-MNHN, Mécanismes Adaptatifs et Evolution, Brunoy, France; University of Southern California, UNITED STATES

## Abstract

Artificial light at night (ALAN) is an increasing phenomenon worldwide. It causes a wealth of biological and ecological effects that may eventually affect populations and ecosystems. Despite the growing concern about ALAN, little is known about the light levels species are exposed to at night, especially for wetlands and underwater habitats. We determined nocturnal irradiance in urban and peri-urban wetlands above and under water, and assessed the effect of cloud cover on the variability of ALAN across the urban gradient. Even in aquatic habitats, cloud cover could increase irradiance beyond values observed during clear full moon nights. We report a negative relationship between baseline irradiance and the increase in irradiance during overcast nights. According to this result and previous studies, we propose that the change in the variation regime of ALAN between the urban center and rural land at its periphery is a usual feature. We discuss the ecological and evolutionary implications of this spatial variation in the urban and peri-urban environment.

## Introduction

Artificial light at night (ALAN) is observed in most ecosystems across the World [1,2]. It is associated with the use of electricity for lighting, and as such has been steadily increasing worldwide for decades with the economic development and growth of the human population [2]. ALAN alters a wealth of biological functions in organisms ranging from physiology (regulation of circadian rhythms, metabolism activity, immune response), to behaviour (activity pattern, resource acquisition, interspecific interactions) [3–8]. The rapid environmental change in the nocturnal environment has elicited warnings about the ecological consequences of ALAN as effects on individuals may propagate up to the population, community and ecosystem level [6,9–11].

With notable exceptions, the increase in nocturnal light intensity is mostly generated by human settlements so that, light intensity is at its highest close to urban centers and decreases outward. Nevertheless, ALAN can be observed across large expanses of land in peri-urban areas and beyond. This phenomenon is caused by skyglow that is due to the scattering of light by particles and aerosols of the atmosphere [12]. Nocturnal spectral irradiance, i.e. the radiant flux of photon per unit area at a specific wavelength, is intrinsically variable because of the lunar cycle and changing weather conditions. However, the increasing use of ALAN drastically changes this natural regime in several ways. Artificial lights are on all year round, thus elevating the mean light intensity level. Cloud cover can amplify skyglow and extends periods of elevated light intensities, effectively disrupting the lunar cycle [13,14], which amplification partly depends on cloud altitude, and overall atmospheric conditions [15]. The effect of cloud cover is now reversed from a shield from lunar light to a reflector of ALAN, thus exposing habitats to a novel regime of illumination at night. The long term consequences of this environmental change are largely unknown.

Studies have mapped ALAN [2] in various areas using remote sensing. Others have focused on the biological effects of ALAN [16–18]. Yet, there has been limited investigation to determine the actual exposure regime to which natural populations are exposed. Even if biological thresholds are determined experimentally, it will be impossible to assess the actual risk for populations if conditions experienced by individuals *in natura* are unknown. This is a key issue given the range of effects ALAN generates. Locally, irradiance depends on the distance to the main light sources and on cloud cover or seasonal factors like the presence or absence of leaves in trees. Therefore, ALAN varies in space and in time, and it is important to find features or regularities in either dimension if we want to predict its long-term effects on natural populations and ecosystems.

We focused on wetlands, which are among the most threatened habitats at the global scale. Some cover large areas, but many are of small size (ponds for instance) and well delineated, especially in areas subjected to high ALAN levels. Many urban areas developed along waterbodies as human settlements were originally situated near rivers, lakes, and wetlands. Wetlands have many functions including water resource, flood protection, and recreation [19–21]. Near or in urban areas they contribute to keep some form of contact between local populations and nature and wildlife. They also host animal and plant communities that have declined because of habitat loss, and their conservation is important for local or regional diversity [22,23]. The multi-functions of wetlands make them habitats of conservation interest that are subjected to various protection statuses. In this regard, it is striking to observe that there has been relatively little interest so far on the short- and long-term consequences of ALAN on the ecological communities of wetlands reflecting probably the need for developing appropriate methods to address this issue [24,25]. Knowledge about the intensity thresholds that cause biological effects is accumulating, including for groups living in wetlands. In many species nocturnal light can alter physiology and development [18,26–30]. Nocturnal light sources lure aquatic insects and alter drift [25,31] or diel vertical migration of zooplankton [24]. There is also evidence that fishes or bacterial community can be affected [32–34]. However, little information is available about the range of nocturnal light intensities aquatic organisms are actually exposed to where they live, but see [35] for aerial measurements. We lack studies that provide *in situ* measurements of ALAN intensities, especially for underwater habitats.

In this study, we assessed the range of nocturnal irradiance wetlands were exposed to across an urban/peri-urban gradient. We took *in situ* measurements of irradiance above water, and measured light transmission in water to estimate light intensities experienced by aquatic organisms. Our second objective was to assess the actual effect of skyglow on the variability of ALAN across the urban gradient to estimate the relationship between intensity and variability of light. Even in aquatic habitats, skyglow can increase irradiances beyond values observed during full moon night without cloud cover. We report here a negative relationship between baseline irradiance and the increase in irradiance due to skyglow. We discuss the ecological and evolutionary implications of the variability of the urban and peri-urban environment at night.

## Methods

### Sampling and measurements

We took *in situ* measurements of ALAN in 26 ponds and 2 river sites in Angers Loire Métropole. Angers is a medium-size city in western France that is crossed by a river. The area, that encompasses the main city (Angers), and its surrounding cities and villages host 275 000 inhabitants. It covers an area of 553 km^2^ but land use is very heterogeneous and includes urban and agricultural land.

Ponds are found in a range of contexts from well-lit residential areas and urban parks to peri-urban agricultural land. Our sampling reflected the expected gradient of exposure of wetlands to ALAN. Geographical coordinate and measurement details for each site are given as supplementary material (S1 Table). On each site, we measured downward spectral irradiance on two night sessions between 23:00 and 02:45. The first session occurred during the full moon under a clear sky (03-05/05/2016). The contribution of skyglow to nocturnal light intensity was expected to be very low. For the second session, measurements were taken on nights under a heavily overcast sky. Irradiances were recorded on 12-14/05/2016, before the first quarter and when Moon elevation was low, and 03/06/2016 during the last crescent when the Moon was below horizon. Although we did not use a survey protocol, we recorded the presence of amphibians, from acoustic or visual contacts, on the sites during measurement sessions.

A common issue of ALAN studies concerns measurement units. Biological photoreceptors respond to spectral irradiance not to intensity per se. The stimulation of a photoreceptor depends on the shape of the emission spectrum of the light source as much as its intensity. Therefore, unidimensional measurements may provide limited information if not related to a spectrum. Many studies now use the very sensitive lightmeter Sky Quality Meter-SQM (Unihedron) to measure nocturnal sky brightness skyglow [7,36,37]. This device is relevant for ecological studies as it measures downward light as organisms experience it, and some efforts have been made to obtain measures of several wavelengths bands with this device [14,37]. Although this caveat has been repeatedly pointed out, there is an ongoing use of units that are not relevant for biological and ecological studies. SQM measurements are given in magnitudes /arcsec^2^ which unit has been developed for astronomy and can be converted in cd/m^2^ (http://unihedron.com/projects/darksky/magconv.php). This luminance unit is not ideal to investigate biological processes. Similarly, luxmeters are used and deliver a single value for light intensity in Lux. Likewise, this unit developed for the human visual system is of limited relevance for other visual systems like dichromats or UV sensitive tetrachromats. Finally, SQM or other lightmeters measure light only over a part of the sky (solid angle < 180°) whereas vector irradiance (solid angle of 180°) or scalar irradiance (all directions) may be more important for organisms. In this regard, hemispherical photography using fish eye lenses, which can be fitted to digital single-lens cameras, have been developed to measure full sky irradiance [38–41]. Thus, devices like SQM and luxmeters, if they can provide information in some cases, are not well-suited to assess the effect of ALAN on a broad range of organisms, especially for visual tasks in animals [42].

To measure absolute downward irradiance, we used a Jaz spectrometer (Ocean Optics), a 600-μm fiber, and spectralon cosine corrector (CC-36UV-S, field of view: 180°, wavelength range: 200–2500 nm, diffuser diameter: 3900 μm) (Ocean Optics). Spectrum range was 300–700 nm. *In situ* measurements are important because they integrate all factors from global (skyglow) to site-specific configuration of visual barriers. Measuring local conditions is highly relevant to assess the exposure of wetland sites. Furthermore, measuring downward irradiance is more likely to reflect the actual conditions of exposure of photoreceptors than distant measure of upward irradiance that typically cannot measure skyglow. To measure irradiance above water surface, we raised the cosine corrector at two meters and oriented it towards the zenith. This procedure ensured that none of the observers cast shadows on the corrector. We also determined light intensity in 23 sites in water bodies. The 5 remaining sites were fenced or had dried. We did not take measurement under the surface. Instead, we took water samples, brought them back to the lab, and measured spectral transmission across the 300–700 nm range using the same spectrometer that can be fitted to a cuvette holder. The JAZ spectrometer contains a Xenon pulsed light source that sends light to the cuvette through a 600-μm fiber. The cuvette (1-cm light path) was filled with pond water to measure transmission spectra. Transmitted lights reached the spectrometer through a 200-μm fiber. Using this transmission spectrum, we measured wavelength-specific absorbance (in log10 units) and computed the expected transmission at two depths 0.5m and 1m. We then multiplied each transmission spectrum by the irradiance spectrum measured above the water surface to compute the expected downward irradiance at both depths. These depth values are biologically relevant because ponds are usually shallow. Furthermore, absorbance in these habitats is high relative to water absorbance from other water bodies because of absorption by chemical compounds like dissolved organic carbon [43,44] so that most light is filtered within a few meters. Finally, many organisms exploit this depth range to seek light, high temperature or food [45–48]. Raw data from this study are available in S2 Table.

### Ethics statement

No specific permissions were required for these activities. The field studies did not involve endangered or protected species.

### Statistical analyses

We tested the hypothesis that spectral irradiance near human settlements is higher under an overcast sky than under a clear sky. We tested pairwise differences in downward irradiances for the two sky conditions (clear, overcast) at three elevations (above ground, -0.5 m, -1.0 m in water). Because the normality criterion was not met, we used Wilcoxon signed-ranked tests. Our second hypothesis was that the relative contribution of cloud cover to skyglow was higher in darker areas than in brighter areas. Thus, we tested the relationship between the ratio of irradiances (overcast/clear) against downward irradiance (clear) using a linear regression. The former is the response variable and the latter is the predictor which can be viewed like the site-level baseline of irradiance.

## Results

**Fig 1:**
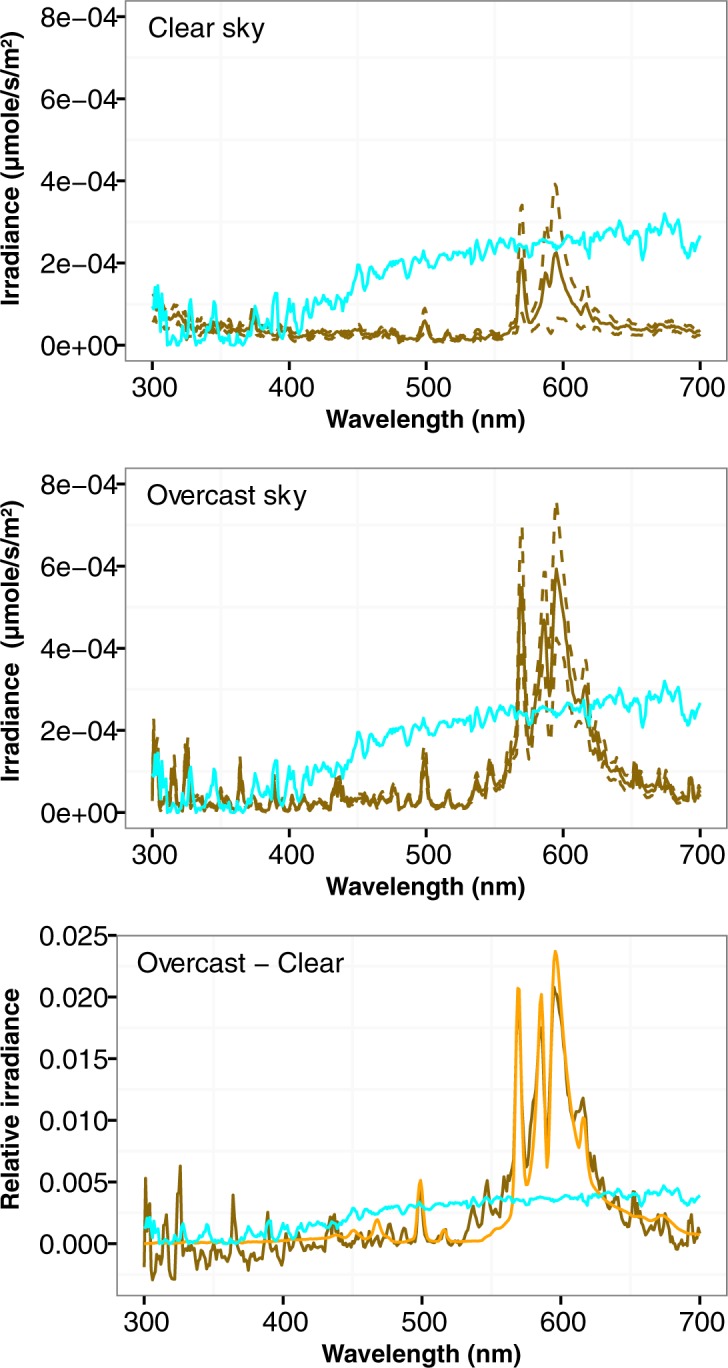
**Effect of cloud cover on nocturnal light intensity (*Up*)** Nocturnal downward spectral irradiance as measured under a clear sky and above the water surface of urban and peri-urban ponds. The brown solid line represents the mean and the dotted brown line the standard error. (***Middle***) Nocturnal downward spectral irradiance as measured under an overcast sky and above the water surface of urban and peri-urban ponds. The brown solid line represents the mean and the dotted brown line the standard error. (***Bottom***). Illustration of the increase of downward irradiance by cloud cover in the emission range of artificial lighting. The brown line represents the difference in irradiance between an overcast and a clear night, the orange line the emission the spectrum of a high pressure lamp. Both spectra have been standardized so that their shape can be visually compared. For all panels, the cyan line represents the downward irradiance of a clear sky during full Moon on 20/07/16 at 1:15 in a rural area (0.604°W, 47.684°N).

Irradiance was higher in the center of the urban area and decreased outwards and the intensity decreased with the distance to the city center (S1 and S2 Figs). The general pattern was affected locally by point light sources in the surroundings of the site. The irradiance spectrum under an overcast sky, averaged for all sites (Fig 1 upper and middle panels), dramatically differed from the lunar spectrum as measured during a full moon night (20/07/2016) under a clear sky in a rural area located about 25 km north of Angers. Furthermore, the change in downward irradiance that we observed between the clear and the overcast night (Fig 1 lower panel) was clearly due to artificial lighting as the spectrum finely matched the emission spectrum of a high pressure sodium light measured under a lamp in the city lamp (Fig 1 bottom panel).

**Fig 2:**
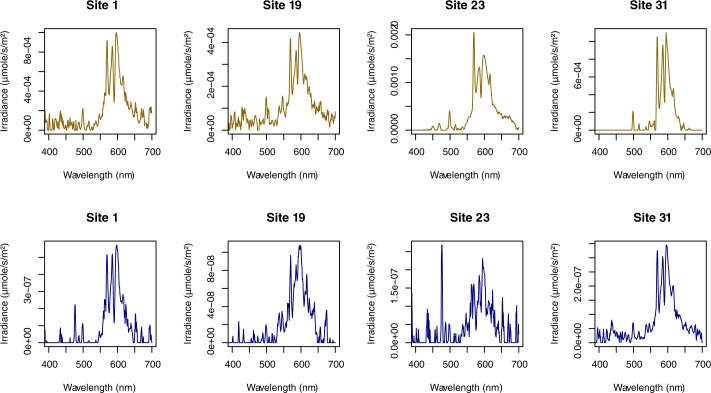
Spectral downward irradiance above water (brown) and below 1 m (blue) in 4 urban and periurban ponds. Irradiance (μmole/s/m^2^) was measured in the 300–700 nm range under an overcast sky conditions.

**Fig 3:**
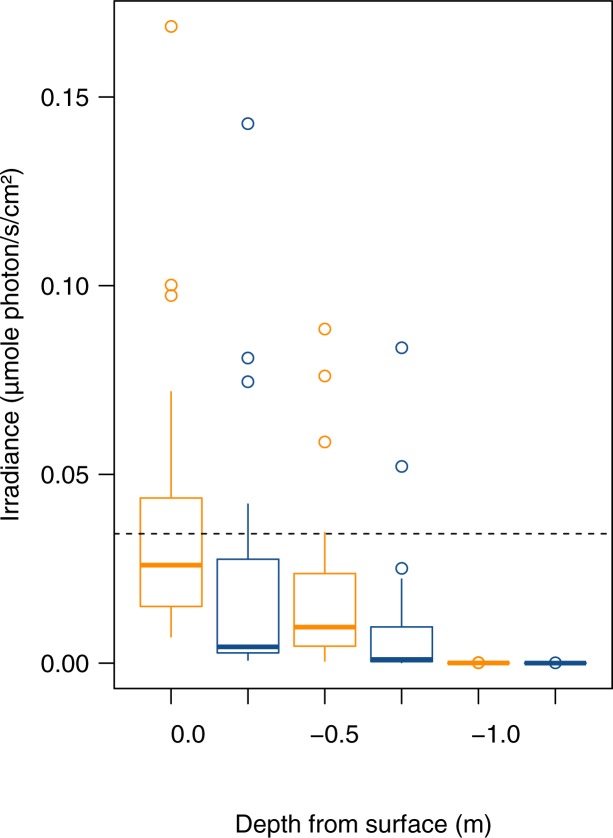
Effect of cloud cover on nocturnal light intensity in 23 freshwater urban and periurban sites. Downward irradiance (μmole/s/m^2^) in the 300–700 nm range measured under overcast (orange) and clear (blue) sky conditions at three elevations (above water, and underwater at -0.5 m, -1.0 m). The horizontal line shows for comparison the downward irradiance measured during a full moon and clear night on 20/07/2016.

Fig 2 shows the effect of water absorbance on the downward irradiance spectra. Even if light intensity is reduced by several orders of magnitude, the spectral signature of artificial lighting is still clearly observable. This result was expected as water absorbance was low in the wavelength range of ALAN (S3 Fig). Consequently, i*n situ* measurements showed a significant increase in downward irradiances under an overcast sky both above and underwater (Fig 3, Wilcoxon signed ranked test for the 3 elevations, p < 0.05). Downward irradiance of the clear moonless nights exceeded the full moon irradiance night in 5 ponds (21.7%) when measured above water, in 2 ponds (8.7%) when measured at -0.5 m and in 0 ponds (0%) when measured at -1.0 m. Under an overcast sky, downward irradiances exceeded full moon irradiance in 11 (47.8%) ponds when measured above the water, in 4 (17.4%) ponds at -0.5 m, and in 0 (0%) ponds at -1.0 m under water.

**Fig 4:**
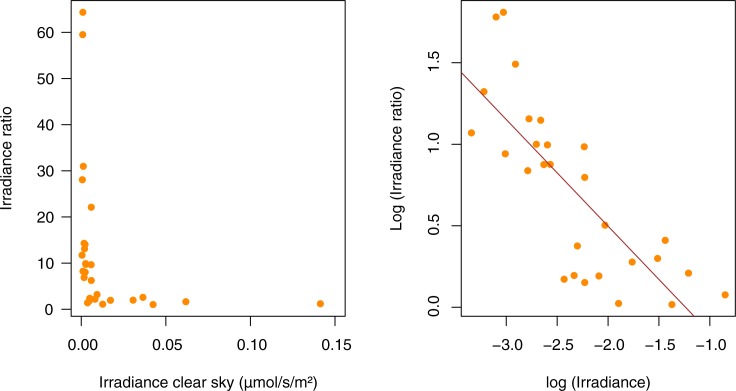
Variation of nocturnal light intensity as a function of cloud cover and baseline level of artificial lighting. The effect of cloud cover is estimated as the ratio of nocturnal irradiances (clear overcast sky over clear sky) against the baseline irradiance of a nocturnal clear sky during the new moon. (left) untransformed data, (right) log-log transformation. The line represents the model of linear regression (r^2^ = 0.612).

Furthermore, relative change in irradiance (irradiance_overcast_/ irradiance_clear_) significantly depended on the baseline (irradiance_clear_) when the relationship was expressed on a log transformed scale (linear regression for above water data: F_1,26_ = 43.68, p<0.001, r^2^ = 0.612, Fig 4). The variability of downward irradiance was therefore dependent on the irradiance measured under the clear sky as the relative increase under an overcast sky was higher was in darker sites than in brighter sites. The values measured in brighter sites show very little fluctuation.

Although we did not survey amphibians using a standard protocol, we detected two amphibian species by their calls in 14 sites during measurement sessions, green frogs (*Pelophylax sp*.) and European tree frogs (*Hyla arborea*). Palmate newts (*Lissotriton helveticus*) and crested newts (*Triturus cristatus*) were found on one site by visual inspection. This information is only qualitative given the period and the method but it shows that most wetlands were used for breeding by amphibians, a flagship group for many conservation actions, and highly sensitive to disruption by light at night [49–51].

## Discussion

We measured the intensity of nocturnal irradiance in ponds along an urban-rural gradient because the potential impact of ALAN on water bodies and wetlands deserves more attention. In particular, quantification of exposure of organisms to ALAN in aquatic habitats deserves more attention. While there is a very recent study on coastal underwater spectral irradiance in the Gulf of Eilat [49], our work is the first on clouds and freshwater. We observed variation in downward irradiance between sites along the gradient. Logically, peri-urban sites experienced lower light levels than sites closer to the town center. Variability occurred even between nearby urban sites depending on the distribution of light sources and visual barriers in the proximity of the sites (S1 and S2 Figs). As reported by previous studies [12], cloud cover significantly increased irradiance. In urban sites, we measured irradiances above and under water surface which values were as high as those of a full moon night under a clear sky. Irradiance exceeded full moon irradiance in half of the sites when measured above water level, and in about 1 out of 5 sites at 0.5 m below the surface, but in none below 1 m. This depth falls within the typical range used by many organisms in ponds, and near lake shores or river banks [45–48].

Our study shows that aquatic organisms are exposed to biologically relevant ALAN. It has been shown experimentally that high light intensities at night can alter microbial communities [50]. However, such levels of irradiance would be experienced only a few meters away from a lamp, and were much higher than in our study. Organisms are sensitive to much lower irradiance levels, and physiological effects have been detected at intensities equivalent to full moon irradiance [17]. Diel migration of plankton in a lake was also altered down to a 4-m depth at that level of nocturnal light intensity [24]. In amphibians, the activity of two newt species [51] and the breeding of two anuran species [52] were shown to vary antagonistically with the lunar cycle. The common toad *Bufo bufo* capture preys at light levels several orders lower than full moon [53]. Their activity and visual sensitivity expose them to biological effects, some of which have been observed [49–51] [54–56]. Thus, many wetlands located in or around cities around the world, including the aquatic habitat, may be exposed to biologically relevant levels of ALAN. Areas that are restored or created for recreational purpose for local populations may be particularly exposed. In this regard, it is interesting to note that Angers is a medium-sized city where lighting with similar characteristics of many cities (not a large use of LED technology, nor a high frequency of light sources emitting very high or very low intensities). Skyglow is expected to be more intense and spread over larger expanses in and around larger urban areas. In addition to its spectral component, ALAN has a spatial component and a time component. We found that the relative change in irradiance strongly depended on the baseline irradiance, i.e. irradiance measured on a clear night during new moon. The lower the baseline irradiance was, the higher the relative increase under an overcast sky was. In our study, relative irradiance changed much less in sites from the interior of the urban area than in peri-urban sites. These results show that not only the mean value of irradiance changes between sites but also its variance, and that both estimators were negatively related. Both the time and the spatial component varied. In our study the more distant sites to the light sources were the most variable. We predict this situation to occur in many contexts. Owing to its effect on biodiversity, ALAN can be considered as an anthropogenic disturbance to the environment. Accordingly, the study of ALAN would gain to be framed in the theories of disturbance ecology [57,58].

Insight into the understanding of the ecological and evolutionary consequences of ALAN is to be gained by investigating the spatial and temporal distribution of ALAN around urban areas. As a priority, it is essential to measure the regime of ALAN to which natural populations are exposed. In this regard, measuring ALAN *in situ* is important. This approach is complementary to satellite-based measurements. Field measurements can document local downward irradiance, and remote sensing measures *ex situ* upward irradiance. Satellites allow the mapping of light sources at a large scale and can be used to estimate the skyglow contribution to downward irradiance from skyglow [2]. Furthermore, remote sensing cannot measure ALAN in aquatic habitats because some local parameters (terrestrial and aquatic vegetation, water colour) are nearly impossible to assess by these methods, especially for small waterbodies. Yet, the availability of higher spatial resolution and time series with a high frequency of data acquisition using ground sensors may help to obtain more realistic exposure measurements of biodiversity to ALAN [59].

It is equally important to identify the groups and traits most likely to be subjected to evolutionary changes from ALAN. For instance, in vertebrates, individuals may eventually change the environmental cues that regulate circadian rhythms and physiological processes. The weight of photoperiod in regulation may be reduced, as observed in species living at high latitudes [60], and more weight could be given to other environmental cues like temperature [61,62]. According to our measurements, we expect ALAN to represent a greater exposure risk for animal than for plant populations in wetlands and other ecosystems. Direct effects on physiology have been observed in plants [63,64] but higher light levels are necessary than in animals [23,24]. The irradiance levels as those measured in the study area in, and probably in many urban areas maybe too low to directly influence plant physiology across large areas (but see [65]. Vegetation may be indirectly affected by herbivores or pathogens sensitive to ALAN though.

ALAN generates novel disturbance of the nocturnal environment that varies in space and time. By framing this environmental heterogeneity in the theories of disturbance, we will likely gain insight into the ecological and evolutionary responses to ALAN. Acquiring long term series of biologically realistic data of exposure to ALAN in various habitats is a preliminary but crucial step that will allow the modeling of evolutionary processes generated by this novel disturbance factor. Developing such methodological approaches is relevant and important in the view of designing ecological networks, and conservation areas that ensure the coexistence of humans and biodiversity in an increasingly urbanizing world.

## Supporting information

S1 Fig Map of irradiance values measured under an overcast sky during a moonless night. The grey areas represent the built areas in and around Angers. The hydrographic network is represented by blue lines. Irradiance values are given in μW/cm^2^: Dark blue (0–0.025), light blue (0.025-.050), orange (0.050–1), yellow (>1).
(PNG)

S2 Fig Map of irradiance values measured under a clear sky during a moonless night. The grey areas represent the built areas in and around Angers. The hydrographic network is represented by blue lines. Irradiance values are given in μW/cm^2^: Dark blue (0–0.025), light blue (0.025-.050), orange (0.050–1), yellow (>1).
(PNG)

S3 Fig Transmission spectra of water for 6 sites representing the range of variation encountered in our study. (EPS)

S4 Fig **(a-d)** Irradiance spectra measured on all sites under a clear (blue) and an overcast (brown) sky at a night.
(PDF)

S1 Table Database of irradiance measurements. The location of sampling sites, date and time of measurement, and irradiance values under clear and overcast skies during moonless nights are given.
(XLSX)

S2 Table Nocturnal irradiance spectra measured at 28 sites under a clear and an overcast sky. (XLSX)
