# Supplementary figures and images for: Variability of surface and underwater nocturnal spectral irradiance with the presence of clouds in urban and peri-urban wetlands
Source: PLoS One. 2017 Nov 8;12(11):e0186808. doi: 10.1371/journal.pone.0186808 (PMC5695598; doi:10.1371/journal.pone.0186808)

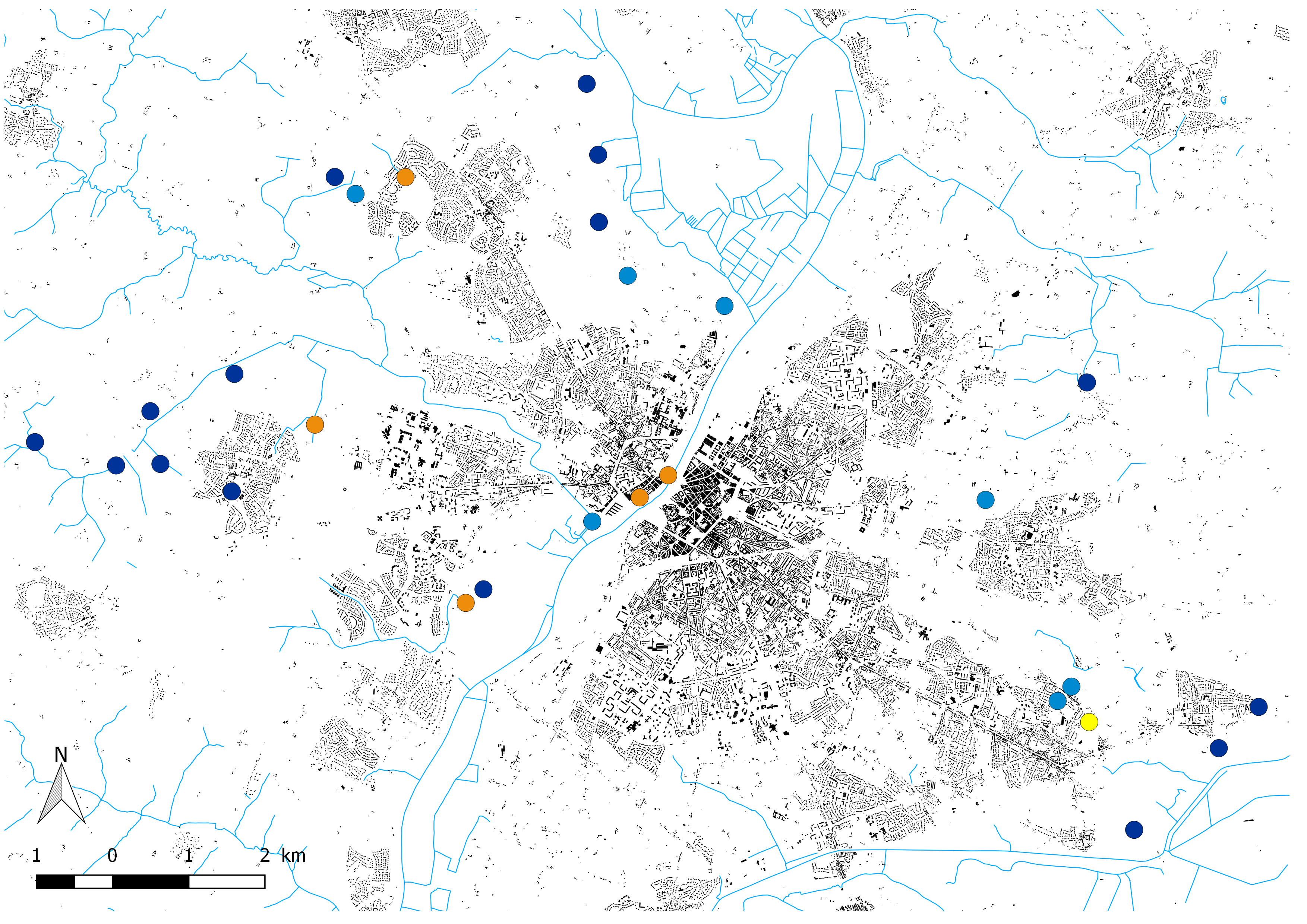

Supplement: S1 Fig — The grey areas represent the built areas in and around Angers. The hydrographic network is represented by blue lines. Irradiance values are given in μW/cm2: Dark blue (0–0.025), light blue (0.025-.050), orange (0.050–1), yellow (>1). (PNG) [file pone.0186808.s001.png]

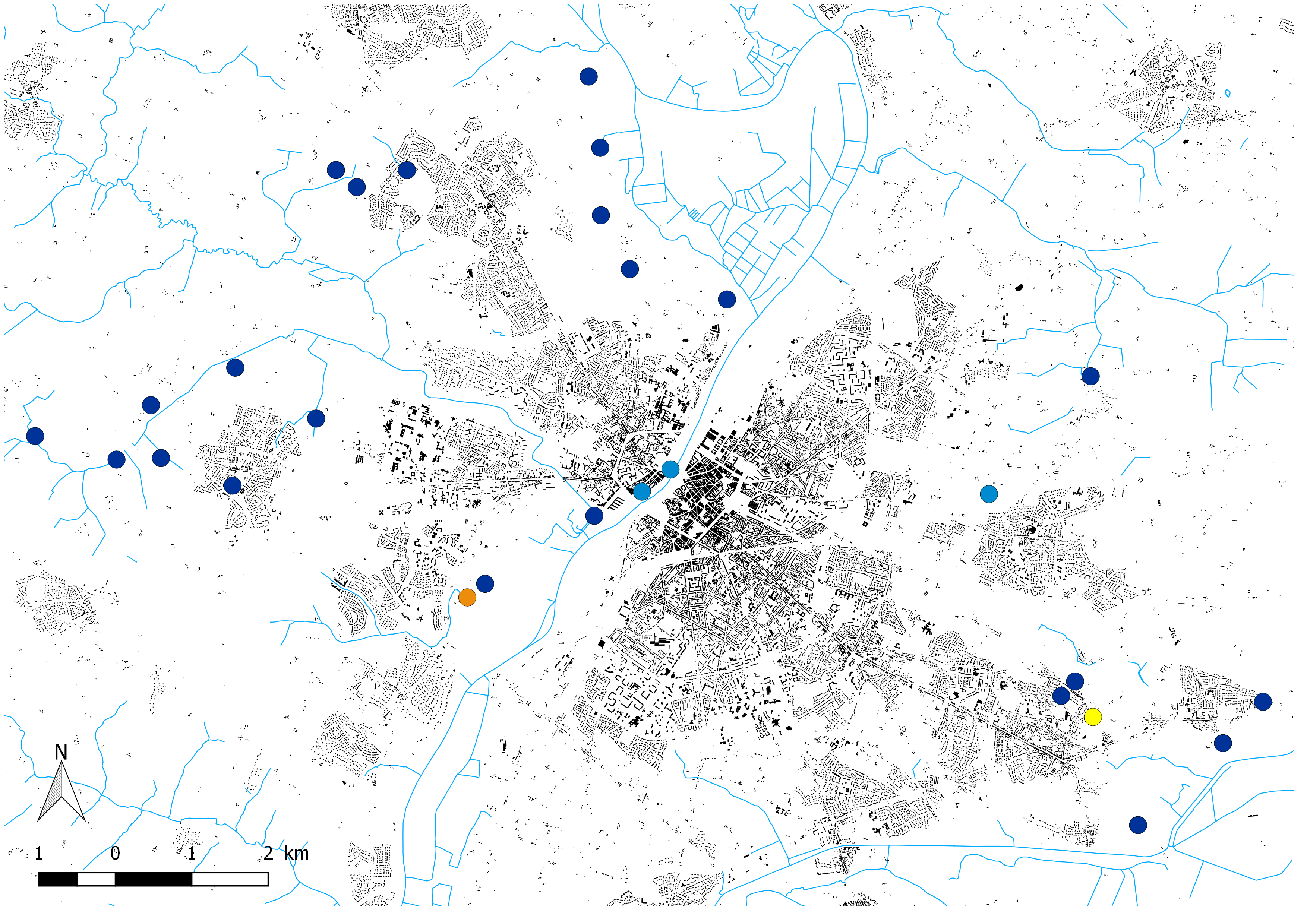

Supplement: S2 Fig — The grey areas represent the built areas in and around Angers. The hydrographic network is represented by blue lines. Irradiance values are given in μW/cm2: Dark blue (0–0.025), light blue (0.025-.050), orange (0.050–1), yellow (>1). (PNG) [file pone.0186808.s002.png]

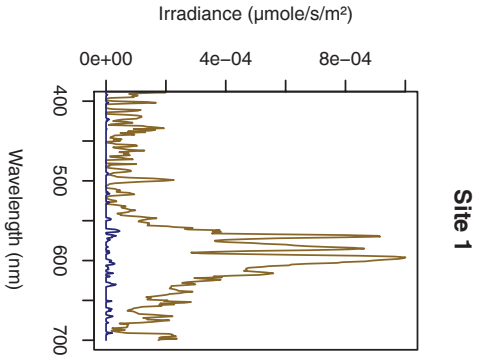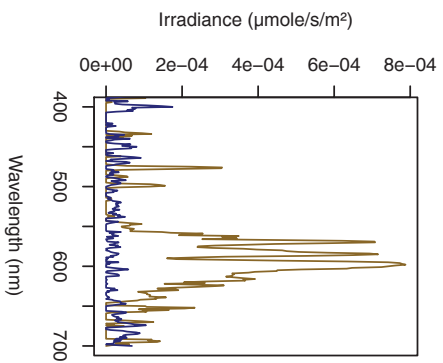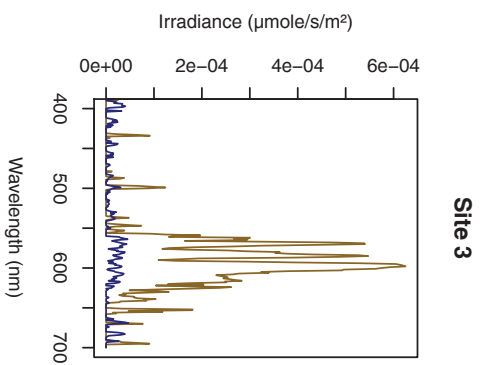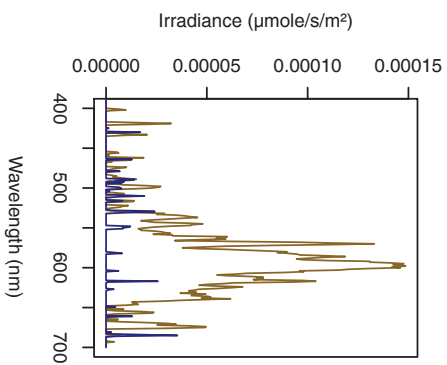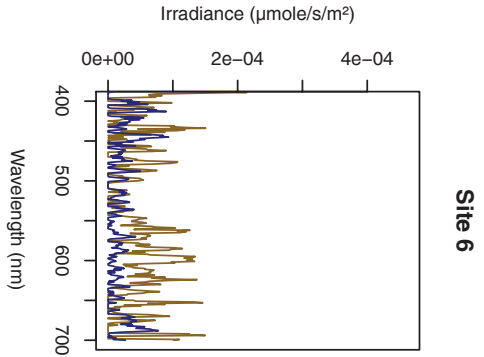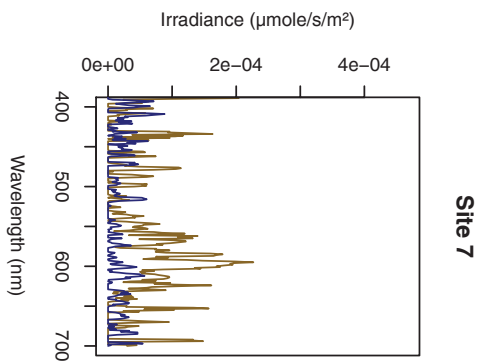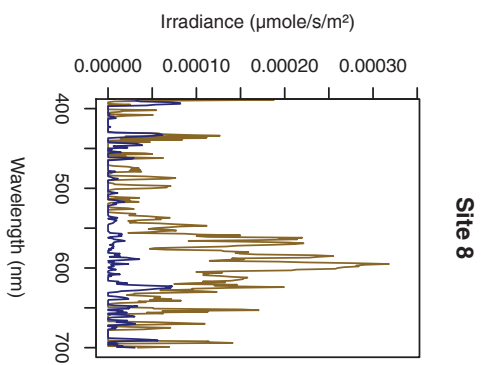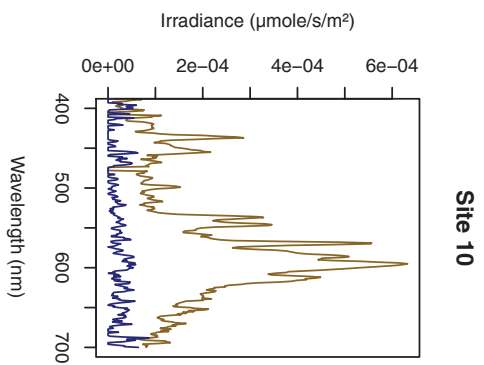

**Site 12**

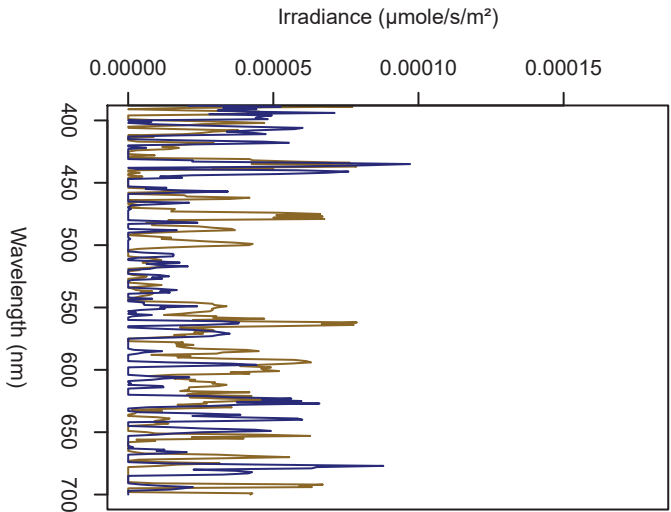

**Site 13**

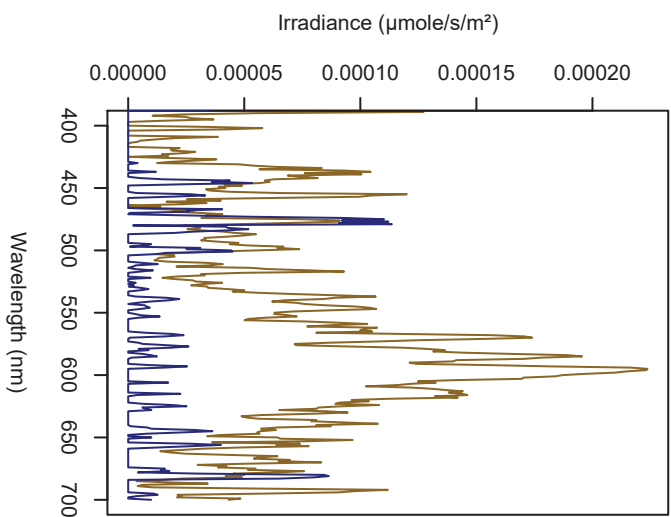

**Site 14**

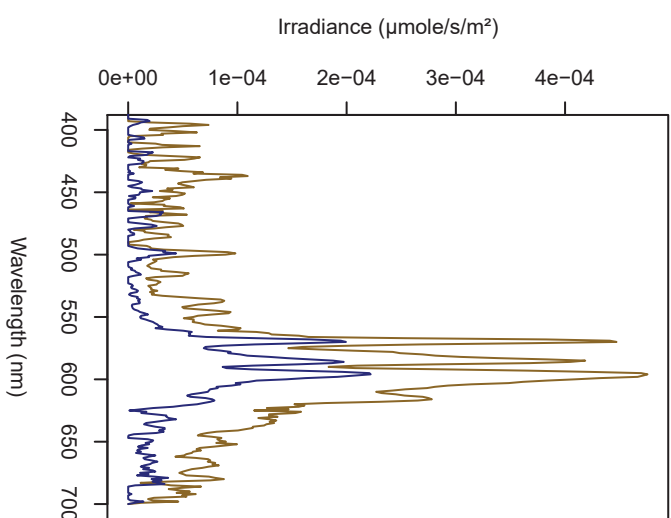

**Site 16**

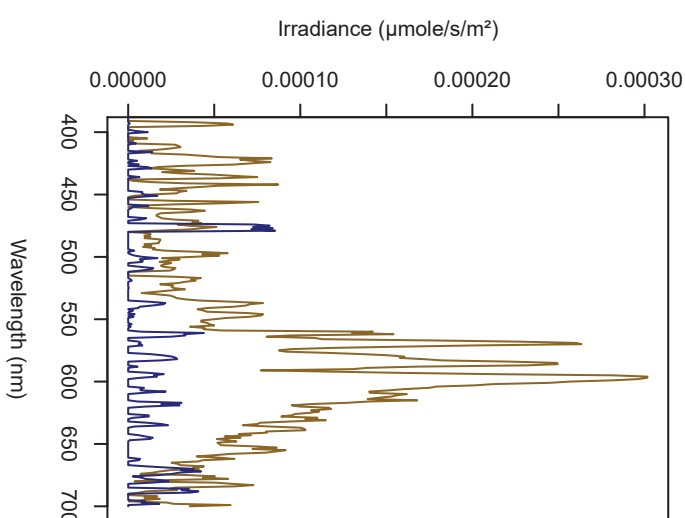

**Site 17**

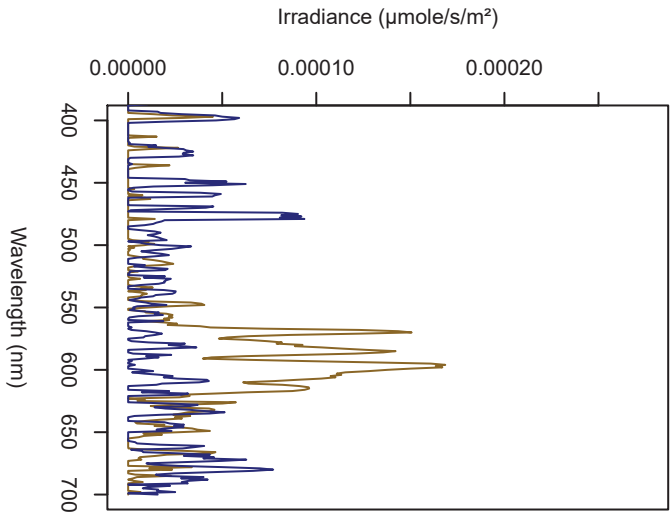

**Site 18**

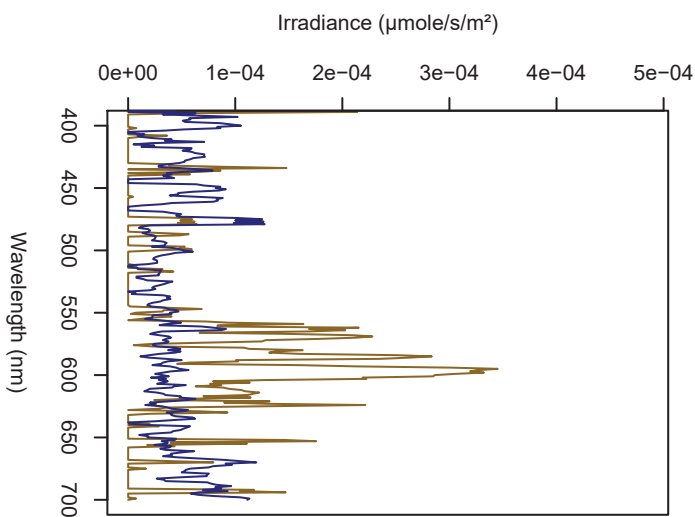

**Site 19**

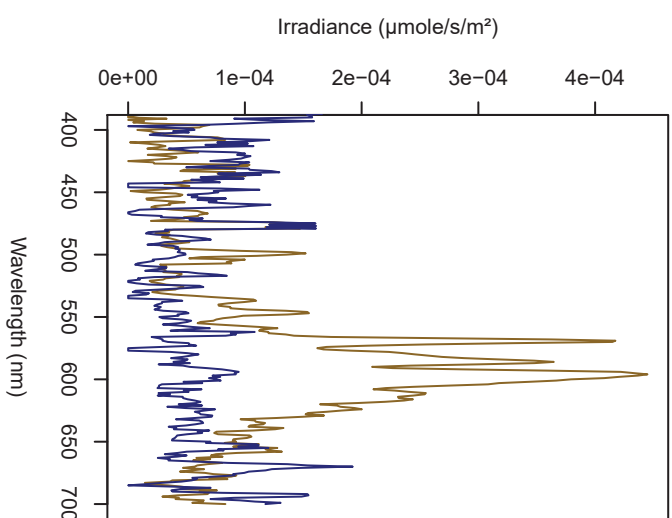

**Site 21**

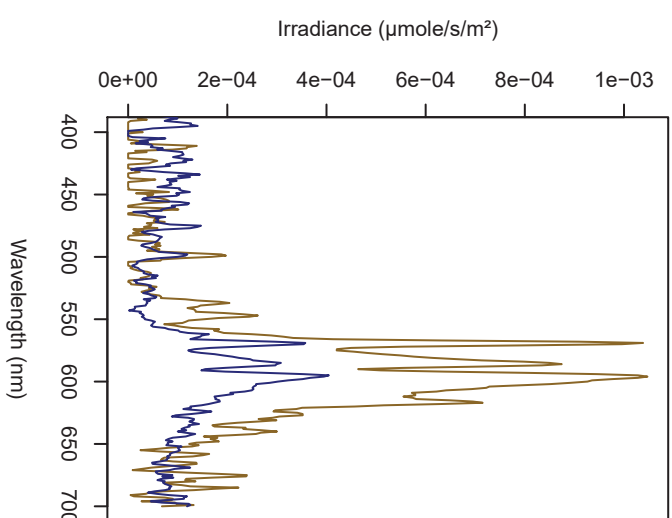

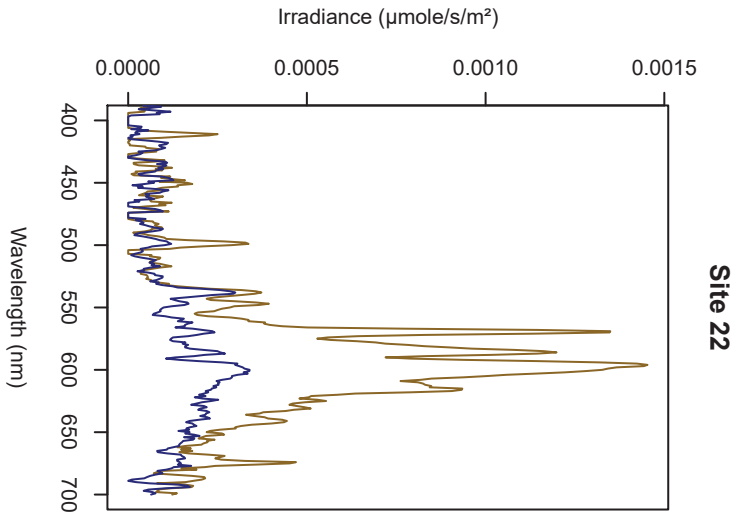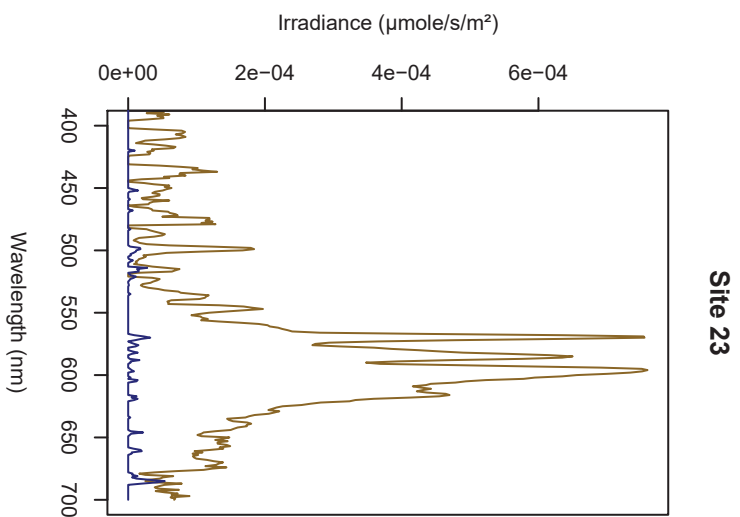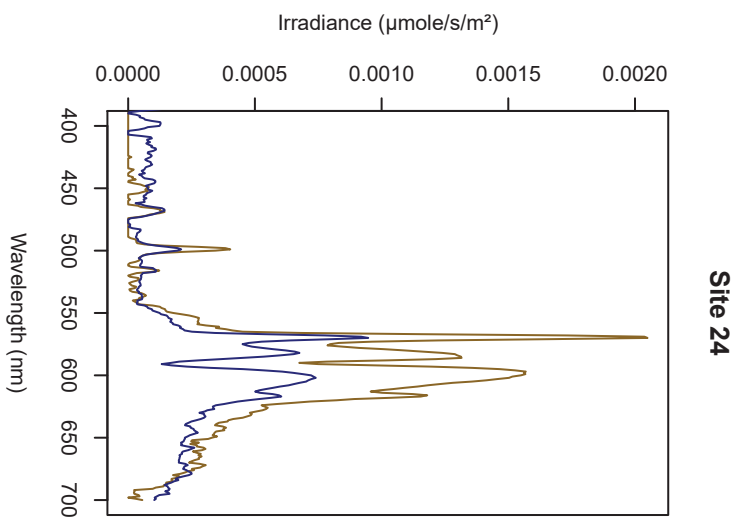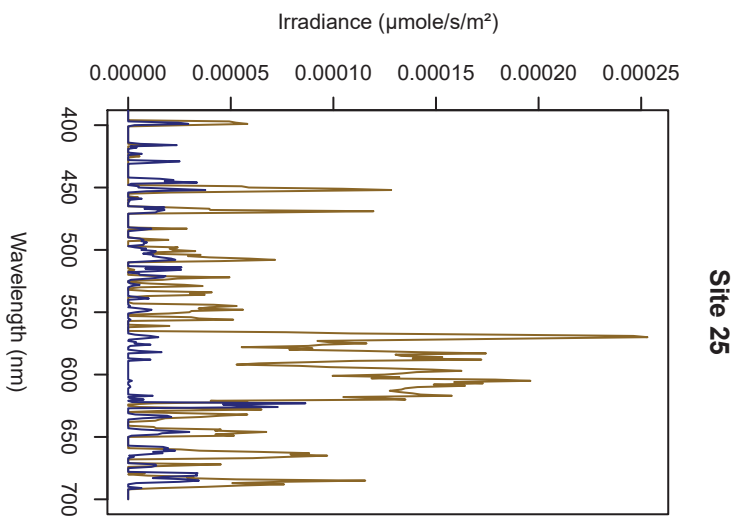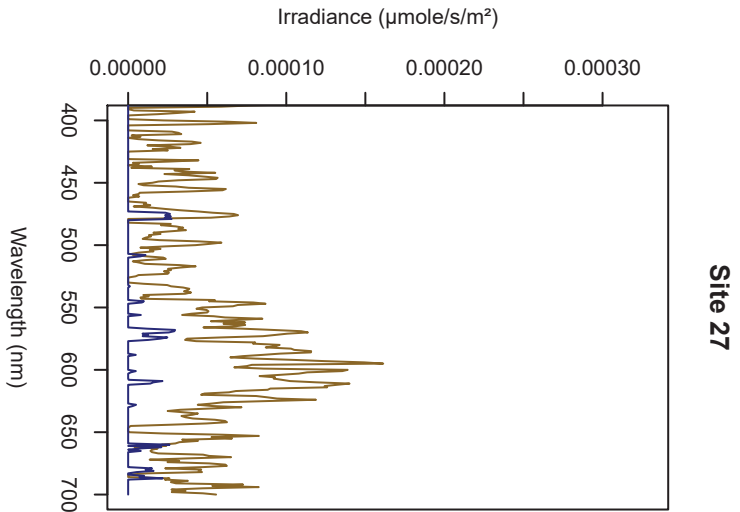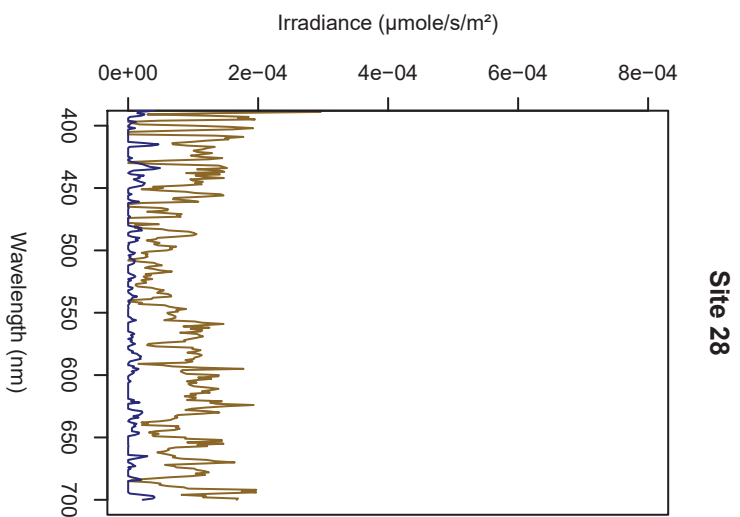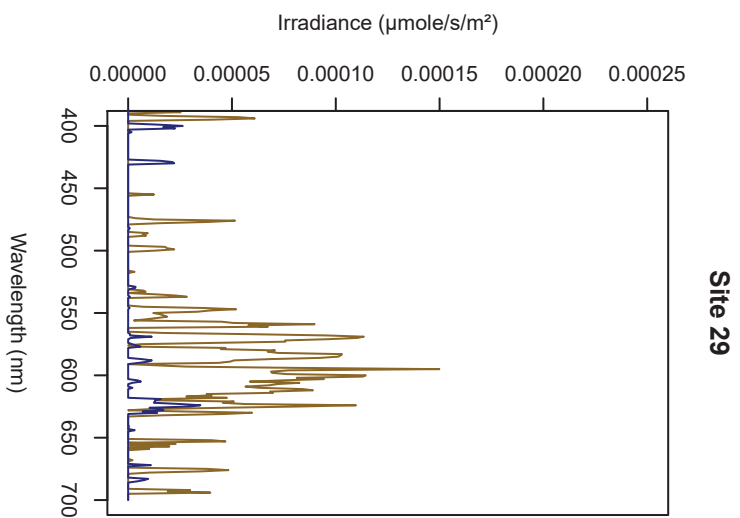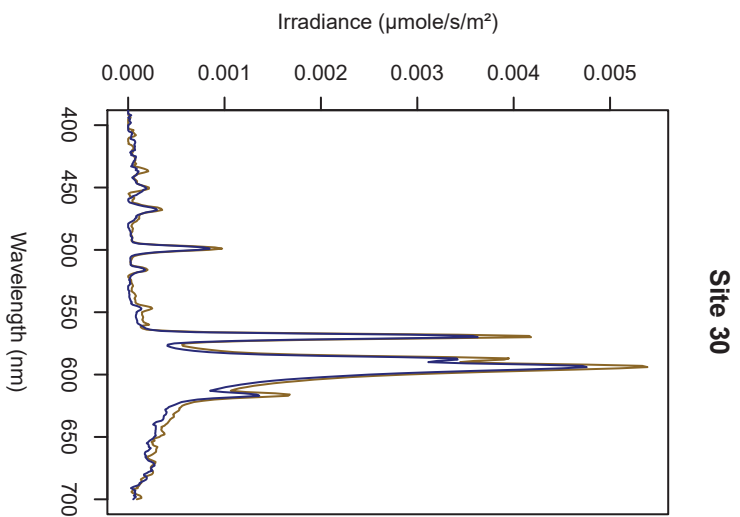

Site 31

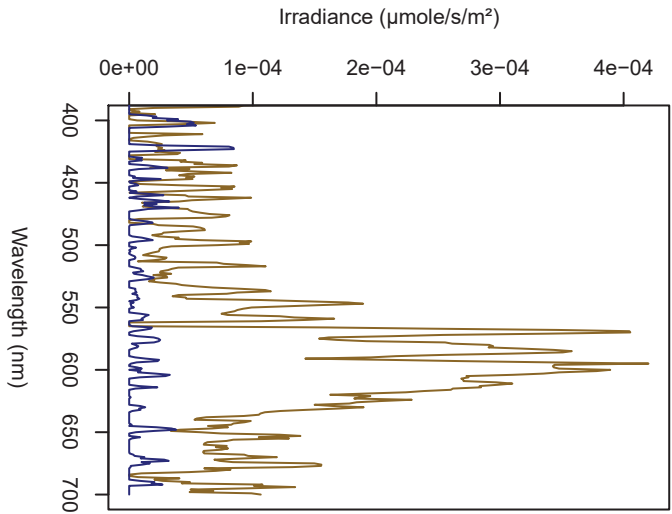

Site 32

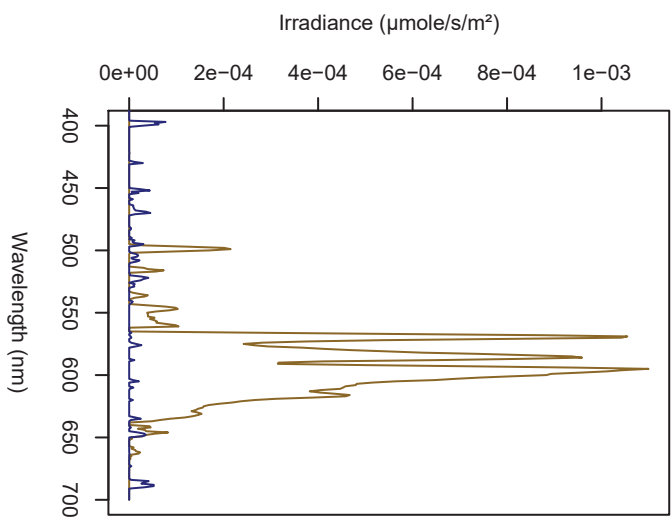

Site 33

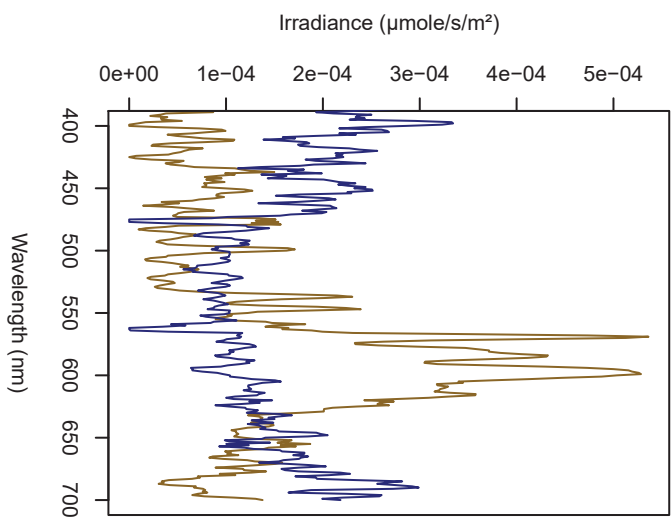

Site 34

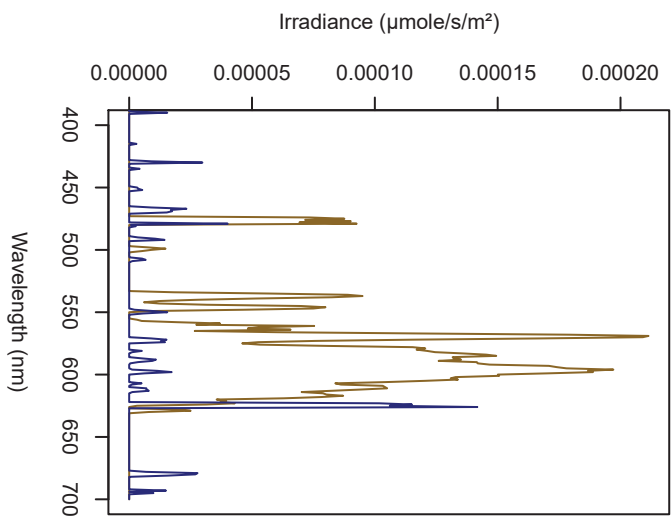

Supplement: S4 Fig — (a-d) Irradiance spectra measured on all sites under a clear (blue) and an overcast (brown) sky at a night. (PDF) [file pone.0186808.s004.pdf]
